# Assessing the carcinogenic and non-carcinogenic health risks of metals in the drinking water of Isfahan, Iran

**DOI:** 10.1038/s41598-024-55615-3

**Published:** 2024-02-29

**Authors:** Maryam Moradnia, Hossein Movahedian Attar, Yaghoub Hajizadeh, Thomas Lundh, Mehdi Salari, Mohammad Darvishmotevalli

**Affiliations:** 1 Department of Environmental Health Engineering, School of Health, Isfahan University of Medical Sciences, Isfahan, Iran; 2 Environment Research Center, Research Institute for Primordial Prevention of Non-Communicable Disease, University of Medical Sciences, Isfahan, Iran; 3 Division of Occupational and Environmental Medicine, Department of Laboratory Medicine, Lund University, Lund, Sweden; 4 Department of Environmental Health Engineering, School of Health, Sabzevar University of Medical Sciences, Sabzevar, Iran; 5 Leishmaniasis Research Center, Sabzevar University of Medical Sciences, Sabzevar, Iran; 6 Research Center for Health, Safety, and Environment (RCHSE), Alborz University of Medical Sciences, Karaj, Iran; 7 Department of Environmental Health Engineering, Faculty of Health, Alborz University of Medical Sciences, Karaj, Iran

**Keywords:** Toxic metals, Non-carcinogenic risk, Carcinogenic risk, Drinking water, Environmental sciences, Risk factors

## Abstract

Metals are significant contributors to water pollution, posing serious threats to human health. This study aims to assess the carcinogenic and non-carcinogenic health risks associated with metals in Isfahan drinking water. Eighty water samples were randomly collected from the city's distribution network between January and March 2020–2021. Inductively coupled plasma Optical Emission Spectrometry was used to measure toxic metals, namely Pb, Cr, Cd, Ni, and As concentrations. Results revealed that the mean concentration of Ni (70.03 µg/L) exceeded the WHO reference value (70 µg/L), while the other metals were below the standard values. The average chronic daily intake order of toxic metals was Ni > Cr > Pb > As > Cd. Non-carcinogenic risk assessment through hazard quotient (HQ) and hazard index (HI) demonstrated that both THI for adults (HQ_ingestion_ + HQ_dermal_ = 4.02E−03) and THI for children (HI_ingestion_ + HI_dermal_ = 3.83E−03) were below the acceptable limit (less than 1). This indicated no non-carcinogenic risk to residents through water ingestion or dermal exposure. However, findings indicated that the ingestion route was the primary exposure pathway, with HQ values for ingestion exceeding HQ values for dermal adsorption. Carcinogenic risk assessment showed that the risk associated with As metal exceeded the acceptable limit (1 × 10^−6^). Therefore, implementing treatment improvement programs and appropriate control measures is essential to safeguard the health of Isfahan City residents.

## Introduction

Ensuring access to safe drinking water and food is critical to sustaining human life, with the overall goal of protecting public health. The growing challenge of water scarcity is a multifaceted threat to global economic development, human well-being, and environmental integrity^1,2^. Contamination of water by various pollutants, including metals and organic/inorganic compounds, adds to these concerns^3,4^. Notably, metals have emerged as important and persistent water pollutants whose levels have increased with rapid economic growth and industrialization^5,6^. This is evident both globally and in regions such as Iran, where soil pollution means an increase in the level of metals in surface and underground water^7–9^. Release of metals into the water can occur through natural processes or human activities, leading to potential human exposure^10^. Understanding this complex interaction between natural elements, industrialization, and water quality is necessary to address the challenges caused by metal pollution in water resources^11,12^.

Human activities significantly affect the availability of metals in the ecosystem. Metals may be present in large amounts through the combustion of fossil fuels, vehicle exhaust, use of fertilizers and pesticides, untreated wastewater irrigation, unprincipled disposal of waste, and atmospheric precipitation caused by various human activities including agriculture, smelting operations, mining, etc. enter the water. It can affect human health by influencing vegetation, food chain, and water quality^13,14^. Due to the unique characteristics of metals such as toxicity, poor biodegradability, and bioaccumulation, they can cause great harm to the environment and human health^15–17^.

Certain metals play crucial roles as structural and catalytic components in proteins and enzymes, contributing to essential metabolic processes within the human body. However, when their concentrations surpass international guidelines, these metals can exert adverse effects on health^18^. Prolonged exposure to elevated levels of heavy metals poses particular risks, as these substances have the potential to accumulate in critical tissues such as the brain, bones, liver, and kidneys. The specific health risks incurred depend on the type of metal and its chemical form, underscoring the importance of monitoring and regulating metal concentrations to mitigate potential harm to human health^19,20^.

The health risk assessment of metals serves as a crucial tool for gauging the overall exposure of a population in a specific region to these elements. This assessment, applied to pollutants, operates on a mechanistic assumption regarding their potential carcinogenic or non-carcinogenic nature^16,21^. This assessment, applied to pollutants, operates on a mechanistic assumption regarding their potential carcinogenic or non-carcinogenic nature^22^. To comprehensively evaluate water quality in a given area, understanding the potential impact of pollutants in drinking water on human health is paramount. While the conventional approach involves a direct comparison of analyzed levels with guideline limits, its reliability in providing comprehensive risk levels for identifying key pollutants is limited. Health risk assessment emerges as an essential method, offering a more nuanced approach to evaluating potential health effects in aquatic environments resulting from exposure to a multitude of pollutants^23^. Widely employed in scientific literature, this method aids researchers in estimating adverse health effects linked to contaminated water exposure^24^.

In Iran, the confluence of population growth, economic expansion, and industrial development has posed a threat to the quality of both surface and underground water, as evidenced by previous studies^16,25,26^. Surprisingly, in Isfahan, a region grappling with these challenges, no prior investigation has addressed the presence of metals in drinking water. This study aims to fill this crucial gap by assessing the levels of five metals—lead (Pb), cadmium (Cd), chromium (Cr), arsenic (As), and nickel (Ni)—in the drinking water of Isfahan City. Additionally, the research focuses on estimating both non-carcinogenic and carcinogenic health risks associated with these metals, taking into account daily water consumption and dermal absorption among the adult population. The findings are anticipated to shed light on metal pollution in Isfahan's water sources, offering valuable insights for residents to adopt protective measures and guiding health professionals in mitigating metal pollution. Moreover, the results will serve as a benchmark for comparisons with other regions in Iran and globally, contributing to a broader understanding of metal contamination in water environments.

## Materials and methods

### Reagents, sampling, and analysis

**Figure 1:**
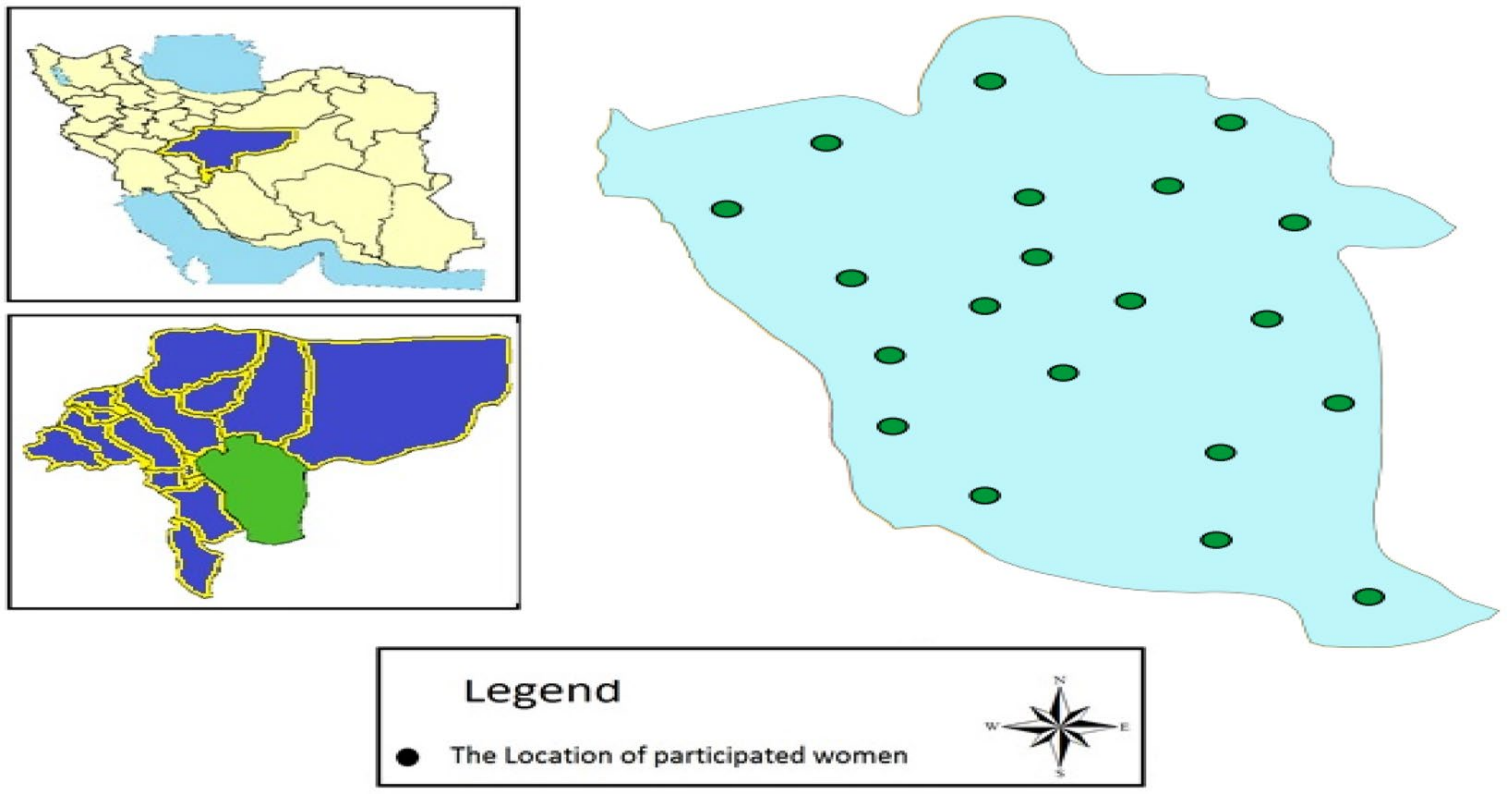
Sampling sites.

In this study, analytical grade HNO_3_ prepared by Merck company was used. Deionized water was used to prepare the solution and also for dilution purposes. All the glass containers were washed with 1% HNO3 and dried in the oven at 105 °C, and finally, the bottles were rewashed with deionized water. A total of 80 water samples were collected from different places along the distribution network between January and March 2020–2021 to measure the levels of toxic elements including Pb, Cr, Cd, Ni, and As in the drinking water of Isfahan City. Figure 1 shows the location of the sampling sites.

The samples were taken to the laboratory and were kept at 4 °C until analysis^16^. The concentrations of metals in all samples were measured using ICP-OES (Varian 720/730-ES, USA).

The quality assurance and quality control (QA/QC) assessments were performed to confirm the reliability of the analytical data and to increase confidence in the relevance of obtained responses. Accordingly, the linear regression gave a good fit (R^2^ ≥ 0.98) with high precision (≤ 13.2% RSD). The limit of detection (LOD) and limit of quantification (LOQ) were based on the signal-to-noise ratio of 3 and 10, respectively. LOD and LOQ for various elements were determined as follows: For Cr: 0.15 µg/L and 0.46 µg/L, Pb: 0.8 µg/L and 2.4 µg/L, Ni: 0.3 µg/L and 0.91 µg/L, As 2 µg/L, 6.2 µg/L, Cd: 0.22 and 0.72, respectively. The R^2^ and precision (% RSD) were obtained as follows: Cr: 0.99 and 7.2%, Pb: 0.98 and 8.4%, Ni: 0.99 and 7.1%, As: 0.99 and 6.7%, Cd: 0.99 and 6.3%, respectively. This study received approval from the relevant institutional review committee, affirming its ethical foundation. Additionally, all methods adhered to the applicable guidelines and regulations.

### Spatial distributions

Spatial analysis of the heavy metal was conducted by ArcGIS software (Version 10.3) based on inverse distance weighting (IDW) interpolation. The method make raster layers that display the average concentration distribution of the targeted pollutants in study area^27,28^.

### Health risk assessment

The assessment of health risks associated with each metal typically relies on estimating the extent of risk and categorizing it as either a carcinogenic or non-carcinogenic health hazard^29^.

#### Analysis of non-carcinogenic risk

In assessing metal contamination and the potential non-carcinogenic and carcinogenic risks associated with ingesting and absorbing metals through the skin via the consumption of drinking water from Isfahan City's distribution network, hazard coefficients (HQ), hazard index (HI), and incremental lifetime cancer risk (ILCR) were employed for the adult community under investigation. The ensuing equations [Eqs. (1) and Eq. (2)] are derived from the guidelines of the Environmental Protection Agency (USEPA)^30^. for estimating chronic daily intake (CDI) via the routes of ingestion and dermal absorption and calculated according to the Eqs. (1) and Eq. (2)^31,32^:

1 $${CDI}_{Ingestion}=\frac{{C}_{w.}DI.ABS.EF.EP}{BW.AT}$$

where: Cw (µg/L): concentration of metals in water, DI (L/day): daily intake average, EF (days/year): annual exposure frequency, ABS (unitless): dermal absorption factor, EP (years): exposure duration, BW (Kg/person): body weight, AT (days): average time*.*

2 $${CDI}_{Dermal}=\frac{{C}_{w.}SA.{K}_{p.}ABS.EF.EP.CF}{BW.AT}$$

where: SA (cm^2^): available skin contact area, CF (L/cm^3^): conversion factor, and Kp (cm/h): permeability coefficient.

Table 1 presents input assumptions and associated values for the computation of chronic daily intake through dermal absorption and ingestion. HQ for each toxic metal was determined by comparing the calculated Average Daily Intake (ADI, mg/kg/day) of a specific metal, whether ingested through contaminated water or absorbed dermally, with the Reference Dose (RfD) for individuals.

**Table 1:** Parameters and input assumptions for exposure assessment of metals through ingestion and dermal routes^20,33^.

Parameter	Values				Probabilistic distribution
Metal concentrations (Cw, µg/L)	Dermal adsorption		Ingestion		Normal
	Adults	Children	Children	Adults	
Daily average intake (DI, L/day)	–	–	0.51 ± 0.14	2.2 ± 0.27	Normal
Permeability coefficient (Kp, cm/h)	Pb: 0.001, Cr: 0.002, Ni: 0.0002, Cd: , As	Pb: 0.001, Cr: 0.002, Ni: 0.0002, Cd: , As	–	–	Point
Skin-surface area (SA, cm^2^)	18,000	8000	–	–	Normal
Exposure time (ET, h/event)	0.2 (0.13, 0.33)	0.2 (0.13, 0.33)	0.2 (0.13, 0.33)	0.2 (0.13, 0.33)	Triangular
Exposure duration (EP, year)	0–6	4	4	70	
Exposure frequency (EF, day/years)	350 (180,365)	350 (180,365)	350 (180,365)	350 (180,365)	Triangular
Conversion factor (CF, L/cm^3^)	0.001	0.001	–	–	Point
Body weight (BW, kg)	16 ± 3.8	70 ± 13.6	16 ± 3.8	70 ± 13.6	Lognormal
Average time (AT, day)	25,550	1460	1460	25,550	Fixed value
ABS	0.001	0.001	0.001	0.001	

The cumulative HQs offer an assessment of the overall potential health risk, denoted as Hazard Index (HI). The HQ calculation related to water consumption is expressed as [Eq. (3)]:

3 $$HQ=\frac{CDI}{RFD}$$

Which: RfD and CDI are quantified in mg/kg-day. Table 2 provides Cancer Slope Factors (CSF) and RfD values for different toxic metals.

**Table 2:** Reference dose (RfD) and cancer slope factor (CSF) for the metals.

Metals	Rdf dermal	Rdf ingestion	CSF mg/kg-day
Pb	0.42	1.4	–
Cr	0.015	3	–
Ni	5.4	20	–
As	0.000285	0.0003	1.5
Cd	0.005	0.5	–

Hazard Index (HI) for multiple metals: The comprehensive evaluation of potential non-carcinogenic health effects resulting from waterborne metal exposure involves calculating the Hazard Index (HI) for various metals, as outlined in the methodology by Bamuwamye et al., Huang et al. 2008 studies^34,35^ using the following Equation.

4 $$HI= \sum_{K=1}^{N}HQ={HQ}_{Pb.}+{HQ}_{Cr.}+{HQ}_{Cd.}+{HQ}_{Ni.}+{HQ}_{ As.}$$

The estimated HI values are compared with standard values: there is the possibility that non-carcinogenic effect may occur in inhabitants when HI > 1, while the exposed individual is unexpected to experience harmful health impacts when HI < 1^36^.

#### Analysis of carcinogenic risk

Potential cancer risks from exposure to a given dose of metals in drinking water can be calculated using the ILCR^37^. ILCR is characterized as the incremental lifetime likelihood of developing any form of cancer due to continuous exposure, lasting twenty-four hours a day, to a specific amount of a carcinogen over seventy years. Equation (5) is a widely employed formula for computing the lifetime cancer risk in such scenarios.

5 $${\text{ILCR}}=CDI.CSF$$

In this context, CSF stands for the cancer slope factor, representing the risk associated with an average concentration of one mg/kg/day of a carcinogenic chemical over a lifetime. This factor is pollutant-specific. The acceptable threshold for a carcinogenic element, whether standalone or in a multi-element context, is taken into consideration^38^.

#### Monte Carlo analysis

The deterministic approach in health risk assessment relies upon static single point along with estimated risk's uncertainty whereas the approach of Monte Carlo simulation can decrease the uncertainty by probabilistic analysis of stochastic variables. To this end, the Crystal Ball software (Version 11.1.2.4, Oracle, Inc., USA) was run for performing Monte Carlo analysis, so that probabilistic analysis was done via 100,000 interactions. More details on Monte Carlo simulations was explained elsewhere^39,40^.

### Ethical approval

This study was approved by the Isfahan University of Medical Sciences Ethics Committee, with the ethical code: IR.MUI.RESEARCH.REC.1400.187.

### Consent to participate

Due to the retrospective nature of the study, the need of informed consent was waived by Isfahan University of Medical Sciences Ethics Committee, with the ethical code: IR.MUI.RESEARCH.REC.1400.187.

## Results and discussion

### The descriptive statistical assessment

**Figure 2:**
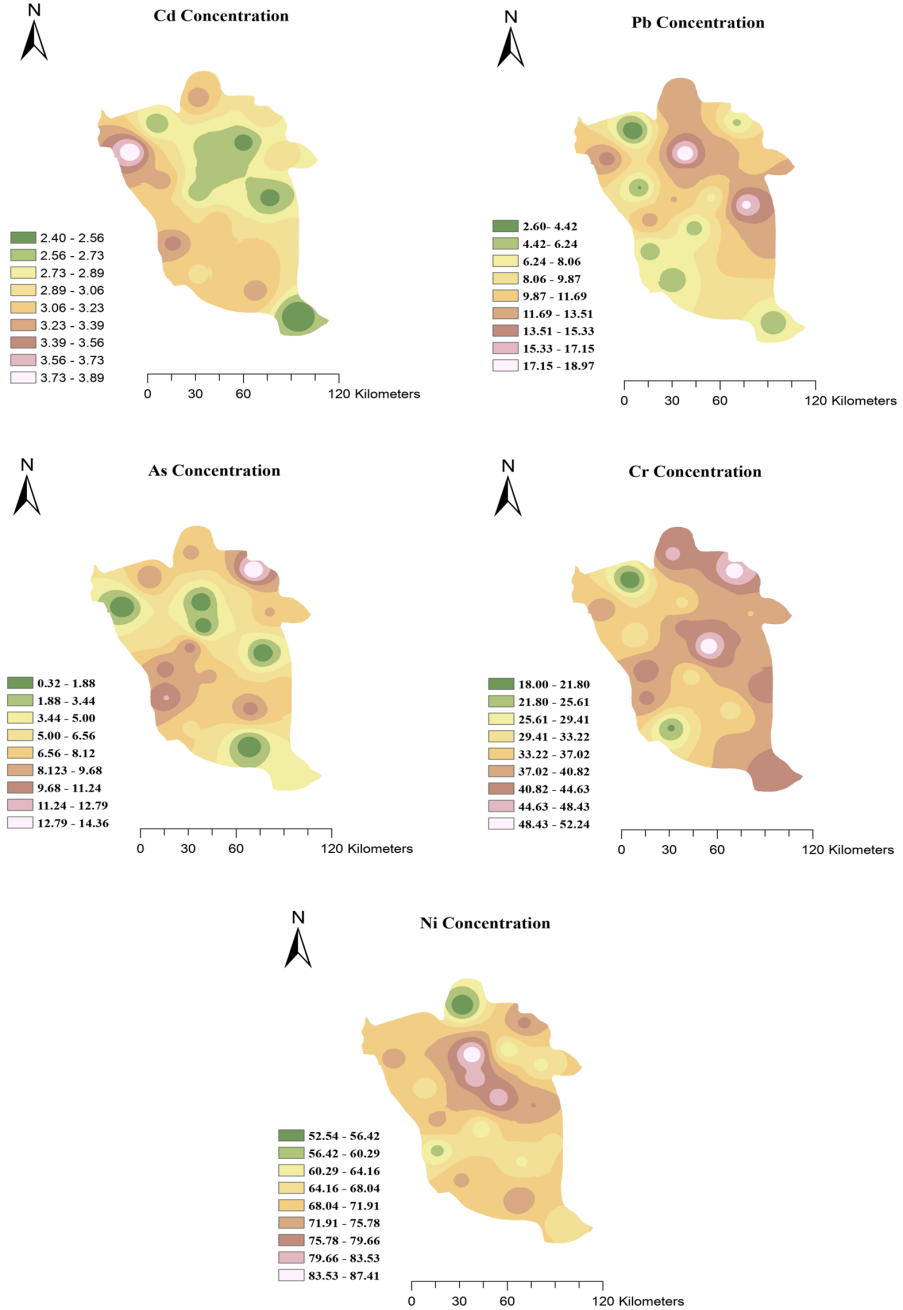
Spatial distribution of Pb, Cr, Cd, Ni, and As elements in study area.

The mean, maximum, and minimum values of the studied metals including Pb, Cr, Cd, Ni, and As elements in the water are given in Table 3. The average concentration of Pb, Cr, and As were lower than the EPA standard values, while their maximum values were higher than the WHO standards. It should be noted that the measured mean concentration of Ni (70.03 µg/L) was higher than the WHO standard (70 µg/L), and also the mean concentration of Cd (2.96 µg/L) was close to the WHO standard (3 µg/L), while the maximum amount of Cd (3.9 µg/L) exceeded the WHO standard. So, the order of the toxicity of the metals according to mean concentrations is as Ni > Cr > Pb > As > Cd. As can be seen in the maps related to spatial distribution of the heavy metal concentration (Fig. 2), Cd metal in the western regions and Pb in the northeastern regions, As in the northeastern and western regions, Cr in the eastern regions and Ni in the central regions have the highest concentrations. Accordingly, the measures is of great importance to identify pollution factors, their control and management based on their spatial distribution features.

**Table 3:** Metal concentrations in the water samples of the study area.

	LOD (µg/L)	Mean (SD) (µg/L)	Maximum (µg/L)	Minimum (µg/L)	Drinking groundwater standard (µg/L)	
					EPA	WHO
Pb	0.8	10.04 (4.60)	18.98	2.61	15	10
Cr	0.15	37.91 (7.69)	52.26	18.11	100	50
Cd	0.05	2.96 (3.02)	3.91	2.44	5	3
Ni	0.3	70.03 (8.77)	87.42	52.54	Not mentioned	70
As	1	6.36 (4.27)	14.36	2.33	10	10

Table 4 indicates the mean, maximum, and minimum, amounts of CDI, as well as total CDI for adults via dermal adsorption and ingestion routes. As it is clear in this table, the highest input values to body was obtained for Ni, Cr, Pb, As, and Cd, respectively. Also, the concentration of metals entering from the oral route is significantly higher than the skin route. Therefore, a special focus in terms of control measures for the oral route should be considered.

**Table 4:** Chronic daily intake (CDI) for the metals via different routes.

	CDI_ingestion_			CDI_dermal_			CDI_total_		
	mean	min	max	mean	min	max	mean	min	max
Pb	3.15E−04	8.17E−05	5.96E−04	1.39E−06	3.71E−07	2.71E−06	3.16E−04	8.21E−05	5.99E−04
Cr	1.19E-03	1.64E−03	5.65E−04	5.42E−06	2.57E−06	7.47E−06	1.20E−03	1.64E−03	5.72E−04
Cd	9.32E−05	7.54E−05	1.22E−04	4.24E−07	3.43E−07	5.57E−07	9.36E−05	7.57E−05	1.23E−04
Ni	2.20E−03	1.65E−03	2.74E−03	1.00E−05	7.51E−06	1.25E−05	2.21E−03	1.66E−03	2.75E−03
As	1.99E−04	4.51E−04	1.02E−05	9.09E−07	4.67E−08	2.05E−06	2.00E−04	4.51E−04	1.23E−05

### Non-carcinogenic analysis

Metal contamination in water can increase human health risks through different routes of exposure. In this study, non-carcinogenic and carcinogenic health risks posed by oral consumption and dermal contact in children and adults were investigated. Exposure to metals occurs through diverse pathways, primarily through ingestion, inhalation, and dermal contact. The assessment of human health risks entails the identification and evaluation of the nature and extent of adverse health effects in individuals exposed to toxic elements in the environment. This process is vital for understanding the potential repercussions of metal exposure on human health and informs measures to safeguard public well-being and environmental quality^41^. In this study, exposure and risk assessments were conducted following the methodology outlined by the United States Environmental Protection Agency (USEPA).

To enhance the robustness of the findings, probabilistic risk assessment was conducted based Monte Carlo method. Incorporating probabilistic elements can provide a more nuanced understanding of uncertainties and variability associated with the studied heavy metal health risks. The potential toxicity of metals to human health is intricately linked to their daily intake. This research specifically investigated metal exposure through drinking water ingestion and dermal absorption. The initial step in evaluating non-carcinogenic effects involves the computation of Chronic Daily Intake (CDI) values. As presented in Table 4, the mean CDI total in mg/kg-day is 3.16E−04 for Pb, 1.20E−03 for Cr, 9.36E−05 for Cd, 2.21E−03 for Ni, and 2.00E−04 for As. Therefore, the mean values of CDI total of metals concentration were obtained as Ni > Cr > Pb > As > Cd.

Table 5 shows the 95th, maximum, and minimum values of HQ and total HQ for adults and children via dermal adsorption and ingestion pathways. As indicated in Table 5, Hazard Quotient (HQ) values for all the examined metals were below 1. This suggests that the health risk assessment for Pb, Cr, Cd, Ni, and As demonstrates a mean HQ_total_ indicative of an acceptable level of non-carcinogenic health risk in all samples collected from Isfahan's water distribution network for both children and adults groups. From the calculation of HQ_total_, it can be concluded that the contribution of the metals to non-carcinogenic health risk for both adults and children was as Ni > Cr > Pb > As > Cd.

**Table 5:** A 95th, minimum, and maximum values of non-carcinogenic human health risks posed by the metals via different routes.

	HQ_ingestion_ (adults)			HQ_dermal_ (adults)			HQ_total_ (adults)		
	95th value	min	max	95th value	min	max	95th value	min	max
Pb	3.15E−04	8.17E−05	5.96E−04	1.39E−06	3.71E−07	2.71E−06	3.16E−04	8.21E−05	5.99E−04
Cr	1.19E−03	1.64E−03	5.65E−04	5.42E−06	2.57E−06	7.47E−06	1.20E−03	1.64E−03	5.72E−04
Cd	9.32E−05	7.54E−05	1.22E−04	4.24E−07	3.43E−07	5.57E−07	9.36E−05	7.57E−05	1.23E−04
Ni	2.20E−03	1.65E−03	2.74E−03	1.00E−05	7.51E−06	1.25E−05	2.21E−03	1.66E−03	2.75E−03
As	1.99E−04	4.51E−04	1.02E−05	9.09E−07	4.67E−08	2.05E−06	2.00E−04	4.51E−04	1.23E−05
HI	4.00E−03	3.90E−03	4.03E−03	1.81E−05	1.08E−05	2.53E−05	4.02E−03	3.91E−03	4.06E−03

	HQ_ingestion_ (children)			HQ_dermal_(children)			HQ_total_ (children)		
	95th value	min	max	95th value	min	max	95th value	min	max
Pb	2.14E−04	5.16E−05	9.60E−04	1.28E−06	4.71E−07	3.51E−06	2.15E−04	5.21E−05	9.64E−04
Cr	2.20E−03	8.90E−04	5.99E−04	5.75E−06	3.47E−06	8.74E−06	2.21E−03	8.93E−04	6.08E−04
Cd	7.30E−05	6.47E−05	2.35E−04	5.12E−07	1.47E−06	9.51E−06	7.35E−05	6.62E−05	2.45E−04
Ni	1.20E−03	1.88E−03	3.47E−03	2.10E−05	8.57E−06	1.25E−04	1.22E−03	1.89E−03	3.60E−03
As	1.12E−04	5.44E−04	2.51E−03	8.59E−07	5.58E−08	4.25E−06	1.13E−04	5.44E−04	2.51E−03
HI	3.80E−03	3.43E−03	7.77E−03	2.94E−05	1.40E−05	1.51E−04	3.83E−03	3.44E−03	7.93E−03

The negligible non-carcinogenic risk to residents' health is evident, given that HI is below 1. The summarized values of HI for metals among the inhabitants in the study area are presented in Table 5.

Moreover, to assess the cumulative non-carcinogenic effects resulting from exposure to multiple metals, the calculated Hazard Quotient HQ for each metal is aggregated and presented as a HI^29^. The average values of HI through ingestion and dermal absorption as well as total HI were respectively obtained as 4.00E−03, 1.81E−05 and 4.02E−03 for adults and 3.80E−03, 2.94E−05, and 3.83E−03, for children. It shows a negligible non-carcinogenic risk to the health of the residents because the HI value is less than 1 (Table 5).

### Carcinogenic risk analysis

Table 6 gives the carcinogenic risk assessment for adults and children. The As element investigated in the study have the potential to elevate the risk of cancer in humans^42,43^. Consequently, prolonged exposure to even small amounts of this toxic element can contribute to the development of various types of cancer.

**Table 6:** The incremental lifetime cancer risk (ILCR) levels of carcinogenic human health risks (for adults) through dermal absorption and ingestion by drinking water of the study area.

	ILCR		
	95th	Maximum	Minimum
As (adults)	1.86E−03	1.39E−03	2.31E−03
As (children)	3.00E−04	6.77E−04	1.85E−05
ƩILCR	2.16E−03	2.07E−03	2.33E−03

The Cancer Slope Factor (CSF) values for As metal, employed in carcinogenic risk assessment, are detailed in Table 2. The overall exposure of residents was assessed based on the mean Chronic Daily Intake (CDI) values, as outlined in Table 4. The carcinogenic risk assessment for adults and children is then presented in Table 6. For one toxic metal, an ILCR > 1 × 10^−4^ is considered harmful and the cancer risk is troublesome while an ILCR < 1 × 10^−6^ is considered insignificant and the cancer risk can be neglected. The acceptable level of ILCR for exposing multiple toxic metals is 1 × 10^−5^^42,44^. As per Table 6, As metal demonstrated a substantial likelihood of cancer risks (> 1 × 10^−5^). The study results suggest a notable cancer risk to residents due to the toxic metal, primarily through the combined pathways of ingestion and dermal adsorption via the region's drinking water.

## Conclusions

According to the mean concentrations of the metals measured in drinking water, the toxicity order was as Ni > Cr > Pb > As > Cd, and the mean values of CDI total in the adults' group were as Ni > Cr > Pb > As > Cd. According to the findings, the ingestion pathway compared to dermal adsorption is the major route of exposure (HQ values for ingestion (4.00E−03 for adults and 3.80E−03 for children) were higher than HQ values for dermal adsorption (1.08E−05 for adults and for 1.40E−05 children). Results of non-carcinogenic indicated a negligible non-carcinogenic risk to the health of the residents but according to the carcinogenic findings, As elemets had a high chance of cancer risks (1.86E−03 for adults and 3.00E−04 for children). Therefore, implementing treatment improvement programs and appropriate control measures is essential to safeguard the health of Isfahan City residents.

## Data Availability

The material and raw data are available from the corresponding or first author upon request via email.
